# Genetic Diversity and Virulence Factors of *S. aureus* Isolated from Food, Humans, and Animals

**DOI:** 10.1155/2020/1048097

**Published:** 2020-08-27

**Authors:** Roberto Adame-Gómez, Natividad Castro-Alarcón, Amalia Vences-Velázquez, Jeiry Toribio-Jiménez, Abigail Pérez-Valdespino, Marco- Antonio Leyva-Vázquez, Arturo Ramírez-Peralta

**Affiliations:** ^1^Laboratorio de Investigación en Patometabolismo Microbiano, Universidad Autónoma de Guerrero, Chilpancingo, Guerrero, Mexico; ^2^Laboratorio de Investigación en Microbiología, Universidad Autónoma de Guerrero, Chilpancingo, Guerrero, Mexico; ^3^Laboratorio de Investigación en Inmunobiologia y Diagnóstico Molecular, Universidad Autónoma de Guerrero, Chilpancingo, Guerrero, Mexico; ^4^Laboratorio de Investigación en Microbiología Molecular y Biotecnología Ambiental, Universidad Autónoma de Guerrero, Chilpancingo, Guerrero, Mexico; ^5^Laboratorio de Ingenieria Genética, Departamento de Bioquímica, Escuela Nacional de Ciencias Biológicas, Instituto Politécnico Nacional, Ciudad de México, Mexico; ^6^Laboratorio de Investigación en Biomedicina Molecular, Universidad Autónoma de Guerrero, Chilpancingo, Guerrero, Mexico

## Abstract

*Staphylococcus aureus* is a commensal bacterium in humans and animals able to adapt to multiple environments. The aim of this study was to compare the genetic diversity and virulence profiles of strains of *S. aureus* isolated from food (29 strains), humans (43 strains), and animals (8 strains). 80 lipase-producing strains belonging to a biobank of 360 isolates, identified phenotypically as *S. aureus*, were selected. Confirmation of the species was made by amplifying the *spA* gene and 80% (64/80) of the strains were confirmed within this species. The virulence profile of each of the isolates was determined by PCR. The *seA* gene coding for enterotoxin A was found in 53.1% of the strains, the *saK* gene, which codes for Staphylokinase, was amplified in 57.8% of the strains, and, finally, the *hlB* gene coding for *β*-Hemolysin was amplified in 17.2%. The profile of antimicrobial resistance was determined by the Kirby Bauer method showing that the strains from food presented greater resistance to erythromycin (40.7%) and ciprofloxacin (18.5%) while in strains isolated from humans were to erythromycin (48.4%) and clindamycin (21.2%). Also, in strains from animals, a high resistance to erythromycin was observed (75%). The frequency of MRSA was 12.5% due to the presence of the *mec* gene and resistance to cefoxitin. Of the total strains, 68.7% were typed by PCR-RFLP of the *coa* gene using the *AluI* enzyme; derived from this restriction, 17 profiles were generated. Profile 4 (490 bp, 300 bp) was the most frequent, containing a higher number of strains with a higher number of virulence factors and antimicrobial resistance, which is associated with greater adaptation to different environments. In this study, a wide genetic diversity of strains of *S. aureus* from different foods, humans, and animals was found. This demonstrates evolution, genetic versatility, and, therefore, the adaptation of this microorganism in different environments.

## 1. Introduction

*Staphylococcus aureus* is a commensal bacterium that is part of the microbiota of the skin and mucous membranes of humans and some animals. However, when the conditions in the host are adequate for the proliferation of the microorganism, it causes pathologies in humans such as pneumonia, endocarditis, osteomyelitis, impetigo, skin infections, and healthcare-associated infections (HAI) [1]; in animals, it is associated with infections in mammary glands [2], and, in the case of food, *S. aureus* produces a great diversity of enterotoxins that generate food poisoning in humans due to the consumption of these [3]; being a microorganism, it also affects the quality of foods, such as raw meat products [4] and milk and dairy products [5].

The successful colonization of *S. aureus* in multiple environments, different inanimate hosts, or matrices is possible due to the large number of virulence factors that this microorganism uses [6]. *S. aureus* produces a large number of enzymes that promote the virulence of this microorganism, including coagulase, staphylokinase, and *β*-lactamases [7]. Among the virulence factors that have an important role in pathogenicity are adhesins and surface proteins, such as protein A, and, in the particular case of toxins, enterotoxins of *S. aureus* (SE) and *β*-Hemolysin (Hlb) [8].

*S. aureus* has the ability to control the expression of virulence factors according to the environmental conditions in which it is found through a global regulation system known as accessory regulatory gene (Agr) [8] and the sigma factor (*σ*B) [9]. In addition to gene regulation, the adaptation of *S. aureus* in different microenvironments with different environmental, nutritional, and stress conditions could generate the acquisition of genes coding for virulence factors that allow its survival [10]. The mobilization of several genes that code for virulence factors in the same mobile genetic element has been observed; for example, in the bacteriophage *β*C-*φs*, the staphylokinase gene and the complement inhibitor protein are mobilized with enterotoxin genes and Panton- Valentine leucocidin[11]. Also, in the *Staphylococcus aureus* pathogenicity islands (SaPIs), this phenomenon has been observed, mobilizing two genes of enterotoxins along with the toxin gene of toxic shock syndrome (*tsst*-1) and the adhesion protein Bap [12]. Another example is the catabolic mobile element of arginine that is transported together with the methicillin resistance cassette (SCCmec) due to its proximity and which has been related to strains that have specific subtypes of SCCmec [1010]. Transfer of antibiotic resistance genes is common in Staphylococcal species [13]. Resistance against methicillin, lincosamides, macrolides, aminoglycosides, and a combination of these antibiotics has been frequently reported in staphylococci [14].

For this reason, it has been difficult to separate a group of *S. aureus* clones with certain virulence factors that generate disease from those clones that are only commensals. Therefore, molecular typing techniques, such as pulsed-field gel electrophoresis (PFGE) and multilocus sequencing typing (MLST) [15], have been designed, being the PFGE technique the gold standard for the typing of *S. aureus*. However, due to the high costs, other alternatives of molecular biology have been proposed, such as PCR-RFLPs of the coagulase gene (*coa*) or the protein A gene (*spA*) [16, 17], genes conserved in genus and species, respectively. In the case of typing by *spA*, a 100% relationship was found with PFGE [18]. Currently, the restriction of these genes continues to be used to typify strains of *S. aureus* of different origins [19, 20]. However, the rapid transformation of this bacterium generates the need for the study of virulence factors, which could provide information on their function in the dissemination and adaptation of the same clone of *S. aureus* in multiple environments, considering that most studies evaluate genetic diversity in a particular environment. Therefore, the objective of this study was to compare the genetic diversity and virulence factor profiles of strains of *S. aureus* from food, humans, and animals.

## 2. Materials and Methods

### 2.1. Strains

For this study, 80 strains previously identified at genus level were selected by primary isolation in Baird Parker agar, Gram stain, fermentation of mannitol and trehalose, coagulase, and positive catalase from a collection of 360 strains of *S. aureus* isolated from different sources (food, humans, and animals), which were stored at −20°C in 20% glycerol (Merck Millipore Cat# 356352, Germany)/brain heart infusion broth BHI (Oxoid, Cat# CM1135, USA). The production of enterotoxin A had been previously determined by dot blot and methicillin resistance in 34 strains [21, 22]. The lipase production was considered as a metabolic characteristic for the selection of the 80 strains for this study due to their participation in infections in humans and animals, as well as their survival and multiplication in contaminated foods [23]. Another important characteristic of this group of strains is that they were isolated from food, humans, and animals from the same city (Chilpancingo, southern Mexico) and nearby towns.

Strains were cultured in BHI broth and incubated for 24 h at 37°C. The control strains used in this study were *Staphylococcus aureus* ATCC 29231 (*sea*), *Staphylococcus aureus* ATCC 14458 (*seb*), *Staphylococcus aureus* ATCC 19095 (*sec*), *Staphylococcus* ATCC 13563 (*sed*), *Staphylococcus* ATCC 27664 (see), and *Staphylococcus aureus* ATCC 25923 (*coa*, *spa*, *hlB*, and *sak*).

### 2.2. DNA Extraction

Total DNA were isolated from 1 mL of an 18 h broth culture from all the bacterial strains including ATCC strains. Cells were pelleted from the cultures by centrifugation at 10,000 rpm for 10 min, resuspended in 300 *μ*L of lysis buffer (10 mM Tris-HCl y 1 mM EDTA, pH 8.0, lysozyme 1 mg/mL), and incubated at 37°C for 0.5 h or until viscous. DNA from all preparations was subsequently extracted with phenol-chloroform and precipitated with ethanol. DNA samples were dissolved in TE buffer (10 mM Tris chloride-1 mM EDTA [pH 8.0]).

### 2.3. Molecular Identification of *S. aureus*

A final point PCR of the *spA* gene was performed to the selected strains for the molecular confirmation of *S. aureus*, with oligonucleotides described in Table 1. The final mixture of each PCR reaction contained 0.2 mM of each dNTP, 3 mM MgCl_2_, 0.2 *μ*M of the oligonucleotides, 1 U of Taq DNA polymerase (Ampliqon Cat# A112103, Denmark), 5 *µ*L of 10X Buffer, and 100 ng of DNA as template. The PCR protocol started with an initial denaturation of 5 minutes at 95°C, followed by 30 cycles at 94°C for 30 s at, 52°C for 30 s, and 72°C for 30 s, and a final elongation at 72°C for 5 minutes. The electrophoresis of the obtained PCR products was carried out in 2% agarose gels at 80V for 60 minutes. The gels were stained with Midori Green (Nippon Genetics, Cat# Mg04, Germany) and visualized with LED light (Nippon Genetics, Germany).

### 2.4. Identification of Genes Coding for Virulence Factors

Detection of genes *hlB*, *mec*, *saK*, *pvL*, *tsst*-1, *seA*, *seB*, *seC*, *seD*, and *seE* coding for *β*-hemolysin, methicillin resistance region, staphylokinase, Panton-Valentine toxin, toxin of the syndrome of the toxic shock, and enterotoxins, respectively, was carried out by PCR in the final point with the oligonucleotides described in Table 1. The final mixture of each PCR reaction contained 0.2 mM of each dNTP, 3 mM MgCl_2_, 0.2 *μ*M of the oligonucleotides, 1 U of Taq DNA polymerase (Ampliqon Cat# A112103, Denmark), 5 *µ*L of 10X Buffer, and 100 ng of DNA as template. In the case of the amplification of the *mec* gene, a concentration of MgCl_2_ of 5 mM was used. The reaction mixtures were subjected to the following amplification programs: initial denaturation of 5 minutes at 94°C, followed by 30 cycles at 94°C for 30 s at, 52°C for 30 s and 72°C for 30 s, and a final elongation at 72°C for 5 minutes, for the amplification of *mec*, *hlB*, *pvL*, *tsst*-1, *seA,* and *seE* genes. Initial denaturation of 5 minutes at 94°C, followed by 30 cycles at 94°C for 30 s, 52°C for 45 s, and 72°C for 45 s, and a final elongation at 72°C for 5 minutes, for amplification of *saK*, *seB*, *seC*, and *seD* genes. The electrophoresis of the obtained PCR products was carried out in 2% agarose gels at 80 V for 60 minutes. The gels were stained with Midori Green (Nippon Genetics, Cat# Mg04, Germany) and visualized with LED light (Nippon Genetics, Cat# Fg05, Germany).

### 2.5. Expression of *β* Hemolysin

To demonstrate the expression of *β* hemolysin, the strains were cultured by strike on 5% cell blood agar, incubating at 37°C under CO_2_ tension for 24 h. The strains that presented a halo of transparency in the perimeter of the colonies were considered *β*-Hemolytic (*hLB*+). The strains that presented *α*- and *γ*-hemolysis were considered *hlB*−.

### 2.6. Antibiotic Susceptibility Tests

An inoculum of each isolated strain equivalent to 0.5 McFarland scale was swabbed onto the Muller Hinton agar plate (BD Bioxon®, Cat# PA-254032.08, Mexico) and the antibiotic disc was then placed on the plate followed by overnight incubation at 37°C. The inhibition zone was interpreted according to the Clinical Laboratory Standards Institute (CLSI, 2016) [27] guidelines (formerly known as the National Committee for Clinical Laboratory Standards). The tested antibiotics were clindamycin (CC, Oxoid, Cat# CT0064B, USA) (2 *μ*g), cefoxitin (FOX, Oxoid, Cat# CT0119B, USA) (30 *μ*g), rifampicin (RA, Oxoid, Cat# CT0207B, USA) (5 *μ*g), erythromycin (E, Oxoid, Cat# CT0020B, USA) (15 *μ*g), tetracycline (TET, Oxoid, Cat# CT0054B, USA) (30 *μ*g), and ciprofloxacin (CIP, Oxoid, Cat# CT0425B, USA) (5 *μ*g).

### 2.7. Test for Induction of Clindamycin Resistance

An inoculum of each isolated strain equivalent to 0.5 McFarland scale was swabbed onto the Muller Hinton agar plate (BD Bioxon®, Cat# PA-254032.08, Mexico) and the induction test was performed by manually placing a 2 *μ*g clindamycin (CC, Oxoid, Cat# CT0064B, USA) disk approximately 12 mm from a 15 *μ*g erythromycin (E, Oxoid, Cat# CT0020B, USA) disk (measured edge to edge). Induction test results were read at 16 to 18 h using transmitted and reflected light. A blunted zone of clindamycin near the erythromycin disk (D-shaped) indicated a phenotype of inducible resistance (inducible MLSB), resistance to erythromycin, and clindamycin indicated a constitutive resistance phenotype (constitutive MLSB) and sensitivity a clindamycin was defined by the absence of induction of clindamycin resistance in the area close to the erythromycin disk [27].

### 2.8. Molecular Typing of *S. aureus*

To the molecularly confirmed strains as *S. aureus*, the amplification of the *coa* gene was performed by PCR in the final point with the oligonucleotides described in Table 1. The final mixture of each PCR reaction contained 0.2 mM of each dNTP, 3 mM MgCl_2_, 0.2 *μ*M of the oligonucleotides, 1 U of Taq DNA polymerase (Ampliqon Cat# A112103, Denmark), 5 *µ*L of 10X Buffer, and 100 ng of DNA as template. The PCR protocol started with an initial denaturation of 5 minutes at 94°C, followed by 30 cycles at 94°C for 30 s, 52°C for 30 s, and 72°C for 60 s and a final elongation at 72°C for 5 minutes. PCR product was digested for 2 hours at 37°C with 10 U of the restriction endonuclease *AluI* (Thermo Scientific®, Cat# IVGN0446, EE.UU) according to the manufacturer's recommended protocol [16]. The restriction digest fragments were detected by electrophoresis in 2% agarose gels at 70 V for 60 minutes. The gels were stained with Midori Green (Nippon Genetics, Cat# Mg04, Germany) and visualized with LED light (Nippon Genetics, Germany).

### 2.9. Statistical Analysis

The statistical package STATA V. 12 (STATA®, USA) was used to calculate simple frequencies and the Chi square statistical test was used for possible relationships between the frequencies of virulence and antibiotic resistance genes in relation to the origins of isolation of the strains; values of *p* ≤ 0.05 were considered as statistically significant. A logistic regression analysis was performed to estimate differences and a statistically significant relationship among the frequencies of positive strains for *saK*, *hlB*, and *seA*. To compare the frequencies of virulence factors and resistance to antibiotics in MSSA and MRSA strains, Fisher's exact test was used.

The identity coefficient for the PCR-RFLPs technique of the *coa* gene was calculated with the following equation:

(1) $$\begin{array}{c} D=1-\frac{1}{N\left(N-1\right)}\sum_{j=1}^{S}n_{j}\left(n_{j}-1\right), \end{array}$$

where *N* is the total number of strains in the population of the sample, *s* is the total number of types described, and *n*_*j*_ is the number of strains that belong to the type.

## 3. Results and Discussion

In this study, oligonucleotides directed to the *X* region of the *spA* gene, located between the binding domain membrane and the catalytic portion (binding to FC-IgG) of protein A, were used to determine the species of *S. aureus* polymorphic, by the insertion or depletion of repeated sequences in tandem of 24 bp [28]. Of the 80 selected strains, 80% (64/80) were molecularly confirmed as *S. aureus* from the amplification of the *spA* gene. The identified strains were isolated from human nostrils (19/19), milk formula (2/2), and surfaces (5/5). The lowest percentage was obtained in vaginal exudate (1/7) (Table 2).

Protein A is a unique surface protein of the species with evasion functions of the immune system [7]. Other markers used to determine the species were the *nuc* gene (which codes for the stable thermonuclease) [29] and the region of the 16S rRNA gene [19]. However, the amplification of the *spA* gene allows the typing either by a PCR-RFLP technique or sequencing, even from the size of the product generated, indicating differences to rule out clonality of the strains due to the insertion or loss of sequences repeated in tandem in the amplified region. With this technique, 80% of the strains were molecularly confirmed, making comparison difficult because most of the authors commonly use *nuc* amplification for identification. However, the *nuc* gene has been found in other *Staphylococcus* species, both coagulase positive (*S. hyicus*, *S. delphini*, *S. intermedius*, *S. pseudintermedius*, and *S. schleiferi*) and negative (*S. capitis*, *S. caprae*, *S. epidermidis*, *S. warneri*, *S. simulans*, *S. carnosus*, *S. kloosii*, and *S. saprophyticus*) [30].

In the strains confirmed as *S. aureus*, the *seA* gene coding for enterotoxin A was amplified in 53.1%, followed by *tsst*-1 with 9.3% and *seC* with 6.2% and with less frequency the *seD*, *seB*, and *seE* genes with 4.6%, 3.1%, and 1.5%, respectively. In the case of other toxins, the staphylokinase gene (*saK*) frequency was 57.8% and that of *β*-Hemolysin (*hLB*) was 17.2%. In the case of resistance to antibiotics, the frequency of the *mec* gene was 12.5%, classifying the strains as MRSA (Table 3).

Regarding the virulence profiles, the most frequently determined enterotoxin gene was *seA*, similar to that reported by Bayomi et al. [31] and Hoque et al. [32]. On the other hand, several studies have observed the epidemiological transition and an increase in the frequency of the genes of the b (*seB*) [11, 33], c (*seC*) [34], and e (*seE*) [35] enterotoxins. However, in this study, the frequency of the three was low, considering the frequency of *seC*, the closest to *seA*, as reported by Rong et al. [35]. The toxin gene frequency of toxic shock syndrome (*tsst*-1) was even higher than enterotoxins other than *seA*. In this sense, an increase in cases of toxic shock syndrome due to *S. aureus* has been reported in relation to those produced by *Streptococcus pyogenes* [36]. In addition, it is important to note that the higher frequency of *tsst*-1 in relation to the enterotoxins of *S. aureus* has not been observed in other studies. The highest number of *S. aureus seA*+ strains was found in the food group (18/34). In the case of positive strains to the other enterotoxins (*seB*, *seC*, *seD*, and *seE*) were found only in the human group. In this study, the most frequent virulence factor gene was *saK* (57.8%), with a higher number of strains carrying this gene in the human group (*p*=0.001).

It is important to demonstrate the circulation of enterotoxigenic strains in multiple environments, due to the role they play; in food, the production of enterotoxins is associated with the development of food poisoning [2]; in humans, if they prepare food, they could transfer it to them, highlighting the importance of nasal carriers in the epidemiology of the microorganism [22, 31], while in animals, such as cows, enterotoxigenic strains can be transferred from bovine udders by dragging and contaminating milk, the raw material for multiple foods of dairy origin [37].

In addition to the enterotoxins, the genes coding for *β*-hemolysin (*hlB*) and staphylokinase (*saK*) was searched, which, in addition to contributing to virulence, have been described as mutually exclusive. The bacteriophage (*β*C-*φs*) that contains the *sak* and *seA* genes generates non-*β*-hemolytic (*hlB*−) strains when inserted into the *hlB* gene [38]. Therefore, in this study, in addition to determining the presence of the *hlB* gene, the functionality of *β*-hemolysin was proved, for the oligonucleotides are not designed to determine the truncated or complete form of the gene. Therefore, the *β*-hemolysis in sheep blood was determined in the *hlB*+ strains, finding a statistically significant relationship between the nonhemolytic strains and the presence of the *seA* and *saK* genes, determining that they have a high probability of carrying the *seA* gene (*p*=0.056) and the *saK* gene (*p*=0.024) but not necessarily both genes (*p*=0.739). In addition, it has been described that the *seA* and *sak* genes are part of the cluster of evasion of the immune system together with the *hol*, *lytA*, *chp*, and *scn* genes [38], so it is proposed that, in the *seA-saK* strains from this study, the search for cluster genes is associated with immune evasion. When demonstrating the expression of the *hlB* gene, it was found that only 17.18% (11/64) of the strains were *β*-hemolytic and 82.81% (53/64) were *α-* or *γ*-hemolytic. In addition, it was determined if there was a statistical relationship between *β-* and *γ*-hemolytic strains with the frequency of the *saK* and *seA* genes, finding a high frequency of positive strains for the *saK* (*p*=0.056) and *seA* (*p*=0.024) genes in the group of nonhemolytic strains (*hlB*−) (Table 4).

**Figure 1:**
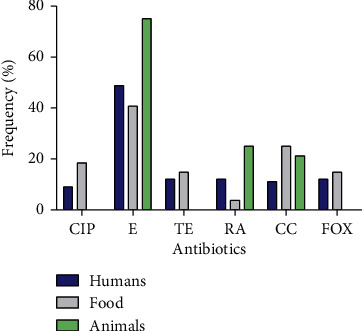
Antibiotic resistance in *S. aureus* strains from humans, food, and animals.

The profiles of antimicrobial resistance showed that the strains from food showed greater resistance to erythromycin (40.7%) and ciprofloxacin (18.5%) followed by tetracycline and clindamycin (14.8% and 11.1%, respectively); the lowest frequency was observed in rifampicin (3.7%). In strains of *S. aureus* from humans, a greater resistance was observed to erythromycin (48.4%), followed by clindamycin (21.2%) and rifampicin (12.1%). A lower resistance was observed to tetracycline (12.2%) and ciprofloxacin (9.1%). Resistance to cefoxitin in both groups was similar (14.8% and 12.1% in food and humans, respectively). Strains from animals showed greater resistance to erythromycin (75%); this is even greater than the other two study groups; no MRSA was found in these strains (Figure 1).

When observing a high resistance to erythromycin, the inducible clindamycin effect was found with a frequency of 1.56%, which corresponds to a strain isolated from the nasal (human) nostrils. Regarding resistance to antibiotics of *S. aureus*, a greater resistance to erythromycin and clindamycin was found in humans. This trend has been reported in clinical isolation [39–41], hands of food handlers [31, 42], and in foods such as chicken [31], fish, and shrimp [35]. As for tetracycline, the frequency of resistant strains was low in both groups, which is contrary to that described by Mekonnen et al. [43] and Asiimwe et al. [34]. This could be explained for the isolates came from animals (cattle) and milk; in this study, there were only 4 strains from cow udders. The frequency of MRSA in this study per group was 14.8% and 12.1% for food and humans, respectively, which is similar to that reported in the hands of chicken handlers (10%), chicken (8%) [31], ready-to-eat food rich in starch (8%) [33], shrimp, and fish (8%) [35]. The highest frequencies are usually from hospital isolates ranging from 35 to 81.4% [11, 40, 41], justifying that in the study the isolates of *S. aureus* were not of hospital origin.

The study of antimicrobial resistance in *S. aureus* has different approaches; in the medical and veterinary area, it is related to therapeutic failure to resolve infections by this microorganism [14, 44]. In the area of food, its participation in the resolution of food poisoning is still under discussion, because it is related to the presence of toxins and not the microorganism, but it has raised food as a vehicle for the transmission of resistant strains [45], which occurs and has been evidenced between animals and humans [46, 47]. In addition, it is important to evaluate resistance profiles, since they change between geographical areas, types of food, and the origin of the strain (whether community or hospital), as observed in what was previously described.

Of the 64 strains confirmed by *spA*, it was possible to typify 68.7% of the strains (45/64) by PCR-RFLP of the *coa* gene, finding 17 restriction profiles (restriction) based on the restriction products generated by the *AluI* enzyme, commonly determined from 2 to 3 fragments of different molecular weight. The product of amplification of the *coa* gene without any cut (profile 1) was considered as a restriction. The most common restriction profile was 4 (490 bp, 300 bp) grouping 7 strains, followed by profiles 2 (400 bp, 300 bp) and 8 (500 bp, 320 bp, and 180 bp) with 5 strains each. According to the origin of the strains, profiles 1 and 2 were only determined in strains of human origin, 14 in strains of animal origin, and profiles 3, 5, 8, 10, 11, 12, 15, and 17 only in strains isolated from food. Profiles 4, 6, 7, 13, and 16 in strains were isolated from both humans and food (Table 5).

A method for the typing of *S. aureus* is the PCR-RFLP technique of the gene that codes for coagulase (*coa*). This enzyme has the function of generating fibrin clots and, thus, participates in the evasion and invasion of the microorganism in the host [7]. The gene is divided into three regions: one cell membrane binding, one polymorphic, and one fibrinogen binding. In the polymorphic region, there may be insertion or loss of tandem repeats of 81 bp. The latter allows typifying the strains, generating different sizes of gene amplification and, therefore, the restriction of the same [16]. The percentages of typing of this gene range from 40 to 100% [16, 19, 28, 43, 48–53]. These typing rates are related to the years in which the isolations were taken, where the percentage is lower (40 and 51%) in studies conducted in recent years [19, 29, 54] compared to those made in the previous two decades, where the percentage was 100% [16, 49–53]. This may be due to the evolution of the strains and the incorporation of mutations in the 3' region of the gene, decreasing the specificity of the used primers, highlighting that the primers described by Goh et al. [16] were used in this study. Another important point is that the study only considered the strains with the amplification of a single product and not the allelic forms, as reported in other studies [16, 19, 48, 53]. The restriction products were generated in this study ranged from 80 bp to 500 bp, which is consistent with has been previously reported [16, 19, 48–53]. It was also considered as a different profile to the PCR product, where there was no cut by the enzyme restriction *AluI*, which was also included in several studies [50–52]. We obtained 17 restriction profiles of 45 unrelated strains, which is similar to that reported by Schwarkopf and Karch [46, 53]; the number of profiles decreases when the strains are isolated from the same origin or environment [29, 54] or have a common characteristic, for example, being MRSA [16, 52]. The diversity of these could be explained by the evolution and adaptation of *S. aureus* to different environments, geographic zones, and even hosts [51]. Even when the number of restrictions is diverse, in most of these studies, one of these restrictions is always the most common, which can be related as the restriction best adapted to the geographical area [29] and even to the guests of the same area; in this sense, it has been described that the restrictotypes, the most common of the studied region, present a greater resistance to opsonization by neutrophils than the less common restrictions [51]. By relating the virulence factors with the restriction profiles, it was found that profile 4 had strains with a higher number of enterotoxin genes. In profiles 7, 11, and 15, strains did not present enterotoxin genes, while in profiles 3, 6, 12, and 13, strains presented the *tsst*-1 gene. In profile 5, there were no strains with the *hlB* or *saK* genes. In profile 4, strains resistant to cefoxitin, clindamycin, ciprofloxacin, erythromycin, and rifampin were found. Also, in profiles 1, 4, 5, 9, 13, and 17, there were strains resistant to cefoxitin. In this study, the most common restriction was 4 (490 bp–300 bp) and it had the most virulence factors, such as *seA*, *seB*, *seC*, *seD*, *hlB*, *saK*, and *mec*, with strains resistant to all antibiotics, except tetracycline, and of combined origin between humans and food, which are associated with a greater adaptation of the strains due to the greater acquisition of virulence factors.

The PCR-RFLP technique of the *coa* gene could be complemented with the amplification data of the *spA* gene; however, it was determined that the power of discrimination of the technique was 0.93, considered within the acceptance range (above 0.8). In other studies in which this technique has been used, the power of reported discrimination was 0.81, 0.99, and 0.80, the first two with strains isolated from milk of cattle with mastitis [48, 49] and the last one of bovine milk [19]. It has been reported that by combining two techniques, for example, PCR-RFLPs of the *coa* gene with ribotyping, the discrimination power is increased from 0.81 to 0.89 [49]; however, in this study, the power of unmatched discrimination was still above that reported. This confirms that the technique of the *coa* gene is easy and reproducible and that it can be used for the typing of strains from different environments.

By grouping the strains according to methicillin resistance, it was observed that strains containing 0 to 2 virulence genes were more frequent in the group of methicillin-sensitive *S. aureus* (MSSA) (83.9%) compared to methicillin-resistant *S. aureus* group (MRSA) (37.5%). Strains carrying 3 or more virulence genes were more frequent with 62.5% in the MRSA group compared to the MSSA with 16% (*p*=0.003). Regarding antibiotic resistance, in the MRSA strains, the antibiotics to which resistance was the highest were erythromycin and ciprofloxacin, with 75% and 37.5% (*p*=0.054), respectively, while in the MSSA strains, the antibiotics to which there was higher resistance were erythromycin and clindamycin, with 42.9% and 10% correspondingly (Table 6).

## 4. Conclusions

In this study, a wide genetic diversity of strains of *S. aureus* from different foods, humans, and animals was found, evidenced by the restriction of the polymorphic region of the gene coding for coagulase, as well as virulence and resistance profiles obtained by PCR at the endpoint and by the Kirby Bauer method, respectively. This demonstrates evolution, genetic versatility, and, therefore, the adaptation of this microorganism in different environments. Remark the importance of its study in different dietary matrices as contaminant and pathogen in animals and humans, as part of the microbiota of the skin and mucous membranes or pathogen in these same sites.

## Figures and Tables

**Table 1:** Oligonucleotide primers used for molecular identification, toxin gene detection, and molecular typing.

Gene (virulence factor)	Sequence (5′-3′)	Size of amplified product (bp)	Reference
*spA* (protein A)	spaF-CAAGCACCAAAAGAGGAA	180–450	[24]
	spaR-CACCAGGTTTAACGACAT		
*coa* (coagulase)	coaF-CGAGACCAAGATTCAACAAG	600–900	[16]
	coaR-AAAGAAAACCACTCACATCA		
*seA* (enterotoxin A)	seaF-TGCAGGGAACAGCTTTAGGC	250	[25]
	seaR-GTGTACCACCCGCACATTGA		
*seB* (enterotoxin B)	sebF-ATTCTATTAAGGACACTAAGTTAGGG	400	[25]
	sebR-ATCCCGTTTCATAAGGCGAGT		
*seC* (enterotoxin C)	secF-GTAAAGTTACAGGTGGCAAAACTTG	297	[25]
	secR-CATATCATACCAAAAAGTATTGCCGT		
*seD* (enterotoxin D)	sedF-GAATTAAGTAGTACCGCGCTAAATAATATG	492	[25]
	sedR-GCTGTATTTTTCCTCCGAGAGT		
*seE* (enterotoxin E)	seeF-CAAAGAAATGCTTTAAGCAATCTTAGGC	480	[25]
	seeR-CACCTTACCGCCCAAAGCTG		
*hlB* (hemolisin *β*)	hlbF-GTGCACTTACTGACAATAGTGC	300	[25]
	hlbR-GTTGATGAGTAGCTACCTTCAGT		
*saK* (staphylokinase)	sakF-ATCCCGTTTCATAAGGCGAGT	260	This work
	sakR-CACCTTACCGCCCAAAGCTG		
*mecA* (methicillin resistant)	mecaF-TCCAGATTACAACTTCACCAGG	180	[26]
	mecaR-CCACTTCATATCTTGTAACG		
*tsst-1* (toxic shock syndrome toxin)	tsstF-CATCTACAAACGATAATATAAAGG	476	This work
	tsstR-CATTGTTATTTTCCAATAACCACCCG		

**Table 2:** Source of isolation and molecular confirmation of strains of *S. aureus* used in the study.

Source	*S. aureus* sub*-aureus*	*spA*+
Animals		
Bovine mastitis	8	4

Humans^&^		
Vagina	7	1
Nostrils^*∗*^	19	19
Nasopharynx	8	8
Hands	9	5

Foods		
Cheeses^$^	15	14
Eggshell	7	6
Food infant	2	2
Surfaces	5	5

^&^Samples were obtained by swabbing or exudate. ^*∗*^Previously isolated on [20]. ^$^Previously isolated on [19].

**Table 3:** Virulence factors of strains of *S. aureus* from different environments.

Virulence factor	Total *N* = 64 *n* (%)	Humans *N* = 33	Foods *N* = 27	Animals *N* = 4	*p* ^+^
Enterotoxins^*∗*^					
*seA*	34 (53.1)	16	16	2	0.702
*seB*	2 (3.1)	2	0	0	0.379
*seC*	4 (6.2)	4	0	0	0.135
*seD*	3 (4.6)	3	0	0	0.228
*seE*	1 (1.5)	1	0	0	0.621
*tsst*-1	6 (9.3)	3	3	0	0.774

Toxins^*∗*^				0	
*hlB*	11 (17.2)	2	7	2	**0.025**
*saK*	37 (57.8)	27	8	2	**0.000**

Antibiotic resistance					
*mec*	8 (12.5)	4	4	0	0.702

^*∗*^Determined by end point PCR. ^+^Determined by Fisher's exact test and square Chi. Values of *p* < 0.05 were considered statistically significant and are marked in table.

**Table 4:** Frequency of the *saK* and *seA* genes in beta (hlb+) and gamma (hlb−) hemolytic *S. aureus* strains.

Virulence factor	*hlb*		*p*
	Positives *n* = 11	Negatives *n* = 53	
*seA*	3 (27.27)	31 (58.49)	0.056
*saK*	3 (27.27)	34 (64.15)	0.024

Values of *p* < 0.05 were considered as statistically significant and are marked in the table. The statistical analysis was performed with Fisher's exact test.

**Table 5:** Restriction profiles of the *coa* gene, virulence factors, and resistance profile of strains of *S. aureus* from different environments.

Profile	*AluI restriction*	Strains	Virulence factor (gen)	Resistance profile	Source
1	800	4	*hlB*, *saK* (1)	Cc (1) Susceptible (2) Fox, E (1)	Humans
			*hlB*, *seA*, *seC* (1)		
			*saK* (1)		
			*saK*, *mec*, *seA* (1)		

2	400 300	5	*saK*, *seA* (1)		Humans
			*saK* (2)	Cip, E (2)	
			*hlB*, *seA*, *seC* (1)	Susceptible (3)	
			*hlB*, *saK*, *seA* (1)		

3	400 150 80	1	*hlB*, *saK*, *seA*, *tsst*-1 (1)	Susceptible (1)	Foods

4	490 300	7	*saK*, *seB* (1)	E (2) Cc (1) Cc, E, Ra (1) Cip, E (1) Fox (1) Susceptible (1)	Foods/humans
			*saK*, *seA*, *seC* (1)		
			*hlB*, *saK*, *seB*, *seC*, *seD* (1)		
			*hlB* (1)		
			*hlB*, *saK* (1)		
			*saK* (1)		
			*saK*, *mec*, *seA* (1)		

5	450 220	2	*mec*, *seA* (1)	Fox (1)	Foods
			---	Susceptible (1)	

6	400 220	3	*saK* (1)	Cc, E (1)	Foods/humans
			*hlB*, *saK*, *seA* (1)	Te, Cip, E (1)	
			*saK*, *seA*, *seE*, *tsst*-1 (1)	Susceptible (1)	

7	500 220	2	*hlB*, *saK* (1)	Susceptible (2)	Foods/humans
			*hlB* (1)		

8	500 320 180	5	*hlB*, *seA* (3)	Te (1)	Foods
			*hlB* (1)	E (1)	
			---	Susceptible (3)	

9	550 400 180	1	*saK*, *mec*, *seA* (1)	Fox, E (1)	Humans

10	400 230 80	1	*hlB*, *saK*, *seA* (1)	E (1)	Foods

11	500 320 80	1	*hlB* (1)	E (1)	Foods

12	400 180 80	1	*saK*, *tsst*-1 (1)	Susceptible (1)	Foods

13	400 200	4	*hlB*, *saK*, *seA* (1)	E (2) Fox, E, Ra (1) Susceptible (1)	Foods/humans
			*saK*, *seA* (1)		
			*saK*, *seA*, *tsst*-1 (1)		
			*saK*, *mec* (1)		

14	400 290 180	1	*hlB*, *saK*, *seA* (1)	E (1)	Animals

15	400 320 80	2	*saK* (1)	Susceptible (2)	Foods
			*hlB* (1)		

16	400 320	3	*seA* (1)	Te (1)	Foods/humans
			*hlB*, *seA* (1)	Susceptible (1)	
			---	E (1)	

17	490 210	2	*saK* (1)	E (1)	Foods
			*hlB*, *mec*, *seA* (1)	Fox, Cip, E (1)	

--- = does not present any virulence gene, CC: clindamycin, RA: rifampin, E: erythromycin, FOX: cefoxitin, TE: tetracycline, and CIP: ciprofloxacin.

**Table 6:** Virulence factors and antibiotic resistance in MSSA and MRSA strains.

	MSSA	MRSA	*p* ^*∗*^
Virulence factor			
0–2 virulence genes	47 (83.9)	3 (37.5)	**0.003**
≥3 virulence genes	9 (16)	5 (62.5)	

Antibiotics			
Clindamycin	9 (16)	2 (25)	0.617
Ciprofloxacin	5 (8.9)	3 (37.5)	0.054
Erythromycin	24 (42.9)	6 (75)	0.133
Rifampicin	5 (8.9)	1 (12.5)	0.567
Tetracycline	6 (10.7)	2 (25)	0.260

^*∗*^Calculated from Fisher's exact test. MSSA, *Staphylococcus aureus* sensitive to methicillin. MRSA, *Staphylococcus aureus* resistant to methicillin. Values of *p* < 0.05 were considered statistically significant and are marked in the table.

## Data Availability

The data used in the study are available on request.
